# Phosphorus metabolism disorders in erythrocytes and lymphocytes among patients with inflammatory breast cancer, infiltrative stomach and colorectal cancer

**DOI:** 10.25122/jml-2022-0048

**Published:** 2022-06

**Authors:** Ivan Ivanovich Smolanka, Irina Yuriivna Bagmut, Michael Ivanovich Sheremet, Oleksii Volodimirovich Movchan, Lyashenko Andriy Oleksandrovich, Ivan Ivanovich Smolanka Jr, Kolisnyk Igor Leonidovich, Oleksandr Volodimirovich Lazaruk, Vitaliy Vasilyevich Maksymyuk, Volodimir Volodimirovich Tarabanchuk

**Affiliations:** 1 National Cancer Institute, Ministry of Health, Kyiv, Ukraine; 2 Kharkiv Medical Academy of Postgraduate Education, Kharkiv, Ukraine; 3 Surgical department No.1, Bukovinian State Medical University, Chernivtsi, Ukraine; 4 Department of Pathology, Bukovinian State Medical University, Chernivtsi, Ukraine

**Keywords:** inflammatory breast cancer, infiltrative stomach cancer, infiltrative colorectal cancer, dysmetabolic processes in erythrocytes and lymphocytes, markers of energy disorder

## Abstract

Energy and plastic potential dysfunction of erythrocytes and lymphocytes among people with inflammatory breast cancer, infiltrative stomach cancer, and infiltrative colon cancer is characterized by a more aggressive clinical course and poor prognosis. We explored the features of energy metabolism and phosphorus metabolism disorders in the erythrocytes and lymphocytes of patients with inflammatory breast cancer, infiltrative stomach cancer, and infiltrative colon cancer as a predicting factor in the course of the disease. 49 people were examined; the 1^st^ group had infiltrative stomach cancer (n=17); the 2^nd^ group had infiltrative colon cancer (n=11); the 3^rd^ group had inflammatory breast cancer (n=21). Glycerol-3-phosphate dehydrogenase activity was 1.8 times reduced (p≤0.005), and the activity of glyceraldehyde-3-phosphate dehydrogenase in erythrocytes of patients with cancer at the main localization increased 2.5 times, compared with normal. Inflammatory breast cancer patients had a statistically significant decrease (p<0.005) in erythrocytes adenosine triphosphate content by an average of 56.5% compared with the normal ratio, and in cases of patients with gastric and colorectal cancer, a decrease of 67%. Excessive use of phosphorus for energy metabolism and adenosine triphosphate production destroys the balance of energetic and plastic potentials of erythrocytes and lymphocytes in inflammatory breast cancer, infiltrative stomach, and infiltrative colorectal cancers patients.

## INTRODUCTION

Energy and plastic potential dysfunction of erythrocytes and lymphocytes among people with inflammatory breast cancer (IBC), infiltrative stomach cancer (ISC), and infiltrative colon cancer (ICC) is characterized by a more aggressive clinical course and poor prognosis [1]. These indicators could be used as markers for choosing treatment tactics and correcting and predicting the disease [2].

When choosing treatment strategies and predicting the course of the disease in inflammatory and infiltrative forms of cancers, using prognosis factors influencing the choice of treatment (the molecular biological composition of the tumor, genetic changes, and immunological response to the tumor) from many factors widely studied and used in clinical practice, we should pay attention to other, less studied, factors as well [3]. So, it is of great interest to examine metabolism in peripheral blood cells, like erythrocytes and lymphocytes [4]. This is because metabolic disorders lead to changes in the normal functioning of erythrocytes and lymphocytes themselves, and further modifications inside the cellular genetic program take place together with metabolic processes [5].

Changes in the shape of erythrocytes are due to the composition adjustments in their membranes; therefore, the plastic processes violation for cancer development. One of the powerful links in carcinogenesis is hypoxia. We can anticipate that dysmetabolism processes in erythrocytes and disruption in their characteristics result from disruption of blood microcirculation, contributing to tumor progression [6]. It was observed that metabolic problems in lymphocytes correlate with the development of their dysfunction [7].

Glycolysis is central to the energy metabolism of erythrocytes. It is known that adenosine triphosphate (ATP) deficiency of the aggregation potential of erythrocytes lowers their deformation and grows the stiffness of the membranes. In addition, proliferation and cytotoxicity capabilities in lymphocytes are impaired [8].

This study aimed to examine the features of energy metabolism and determine the markers of phosphorus metabolism disorders in the erythrocytes and lymphocytes of patients with inflammatory breast cancer, infiltrative stomach cancer, and infiltrative colorectal cancer as a predicting factor of the course of the disease.

## MATERIAL AND METHODS

49 people aged 45 to 65 years were examined from 2017 to 2021. Patients were divided into three groups with different variants of infiltrative forms of cancer. The first group represented the infiltrative form of stomach cancer (n=17); the second group the infiltrative form of colorectal cancer (n=11), and the third group included 21 people with inflammatory breast cancer.

All examined patients had inoperable forms of the tumor process (T3-4, N1-2, M0) at the initial treatment, and the index of proliferative activity of Ki-67 was higher than 50%.

The enzymes we determined are actively used in glycolysis, generating energy and plasticity (intake of glycerol-3-phosphate for synthesizing phospholipids that construct molecular membranes) and adenine nucleotides in erythrocytes and lymphocytes [9]. Therefore, we studied the action of enzymes that use phosphorus (glycerol-3-phosphate dehydrogenase (G3D) and glyceraldehyde-3-phosphate dehydrogenase (GAFD)). The decreased rate of nicotinamide adenine dinucleotide (NAD+), an acceptor of hydrogen particles, decided the movement of GAFD and G3D. The Lowry method was used to determine the protein concentration. The concentration of ATP within the suspension of erythrocytes was assessed by Yaverbaum, and the concentrations of adenosine diphosphate (ADP) and adenosine monophosphate (AMP) by Berg Meyer.

## RESULTS

We found changes in the phosphorus metabolism of erythrocytes and lymphocytes in inflammatory and infiltrative forms of cancers, which allow us to talk about the excessive use of phosphorus for energy metabolism and ATP production.

There was a decrease in the action of glycerol-3-phosphate dehydrogenase (p≤0.005) in erythrocytes, an enzyme that catalyzes the arrangement of glycerol-3-phosphate which acts as an intermediate in the biosynthesis of triglycerides (Table 1). It was reduced by 1.8 times compared to the normal ratio. Furthermore, there was a decline in the glycerol-3-phosphate dehydrogenase activity (p≤0.005), while the activity of glyceraldehyde-3-phosphate dehydrogenase, which characterizes the depth of glucose breakdown, tended to increase (p≤0.05) (Table 1).

**Table 1:** The action of glycerol-3-phosphate dehydrogenase and glyceraldehyde-3-phosphate dehydrogenase in the erythrocytes of patients with cancer.

The activity of enzymes	Stomach (n=17)	Colorectal (n=11)	Breast cancer (n=21)	Normal ratio
Glycerol-3-phosphate dehydrogenase (G3D) in erythrocytes nmol/min•l	6.94±0.45*	5.72±0.50*	5.59±0.25*	12.35±1.18
Glyceraldehyde-3-phosphate dehydrogenase (GAFD) in erythrocytes nmol/min•l	5.49±0.07*	8.67±0.50*	7.42±0.29*	5.18±0.26

* – p<0.05 compared to the normal ratio.

We examined the activity of these enzymes in blood lymphocytes. Table 2 shows the results of G3D and glycerol-3-phosphate dehydrogenase activity in lymphocytes.

**Table 2:** The action of glycerol-3-phosphate dehydrogenase and glyceraldehyde-3-phosphate dehydrogenase in the lymphocytes of patients with cancer.

The activity of enzymes in lymphocytes, nmol/min•l	Stomach (n=17)	Colon (n=11)	Breast cancer (n=21)	Normal ratio
Glycerol-3-phosphate dehydrogenase (G3D)	1.13±0.03*	1.10±0.03*	1.16±0.05*	1.55±0.15
Glyceraldehyde-3-phosphate dehydrogenase (GAFD)	2.55±0.04*	2.95±0.03*	3.29±0.02*	0.69±0.02

* – p<0.05 compared to the normal ratio.

To confirm our conclusions, we observed changes in adenyl phosphates (Table 3). In connection with the above, it was interesting to study the level of adenine nucleotides: adenosine triphosphate (ATP), adenosine diphosphate (ADP), adenosine monophosphate (AMP) – as markers of energy deficiency in cancer patients depending on the location of the process. There was a statistically significant decrease (p<0.05) in erythrocytes ATP content for inflammatory breast cancer by an average of 56.5% compared to the normal ratio. About the same dynamics were observed in cases of patients with gastric and colorectal cancer, by 67%. The groups of patients with stomach and colorectal cancer did not significantly differ.

**Table 3:** The volume of adenyl phosphates depending on the cancer process location.

Indicator Group of the patients	Adenosine triphosphate (ATP), mmol/l	Adenosine diphosphate (ADP), mmol/l	Adenosine monophosphate (AMP), mmol/l
Stomach (n=17)	1.38* [1.27; 1.50]	1.36*^3^ [1.24; 1.51]	1.64*^3^ [1.53; 1.70]
Colorectal (n=11)	1.36* [1.25; 1.51]	1.34*^3^ [1.20; 1.49]	1.62*^3^ [1.51; 1.68]
Breast cancer (n=21)	1.42±0.13*	0.81* [0.70; 0.08]	1.10* [1.03; 1.14]
Normal ratio (n=20)	2.25 [2.10; 2.2]	1.01 [0.93; 1.08]	0.58 [0.52; 0.69]

* – p<0.05 compared to the normal ratio.

There were significant differences in the ATP content of patients with stomach and inflammatory breast cancer. The content of ADP in patients with inflammatory breast cancer tended to decrease, while in patients with stomach and colorectal cancer, there was a statistically significant (p<0.05) increase of 22% compared to the normal ratio and 35% compared with colorectal cancer. For IBC patients, the AMP level, compared with the normal ratio, diminished by 90%. There was an even more pronounced AMP decrease – almost 2.5 times compared to the normal ratio and 42% compared with patients with inflammatory breast cancer.

## DISCUSSION

There was a decline in the glycerol-3-phosphate dehydrogenase activity (p≤0.05) and an increase in the activity of glyceraldehyde-3-phosphate dehydrogenase, which characterized the depth of glucose breakdown, which corresponds to Peiró et al. [10]. Without a doubt, non-inflamed cells display a glycolytic profile, which is in line with past discoveries, with no changes observed in glucose utilization and lactate generation when the extracellular glucose was lifted when generalizing oncologic disease. Usually, transcendent utilization of glycolytic phosphometabolites in energy generation forms, *i.e*. glycolytic ATP synthesis, may increase within the cell required for 2.3-bisphosphoglycerate, which provides oxygen exchange to tissues. Glycolysis could be a central metabolic pathway utilized by all cells for the oxidation of glucose to produce energy as ATP and intermediates among cancer patients [11].

The main localization of GAFD activity increased 2.5 times than the norm in the erythrocytes of cancer patients. Our results showed that cancer patients had increased use of phosphate in glycolysis, almost the same as Li et al. [12], who identified the high metabolic rate of breast cancer patients driven to expand free radical production in the body, approaching an extensive amount of GAFD.

The general tendency to diminish the action of glycerol-3-phosphate dehydrogenase in erythrocytes and lymphocytes was established, which agrees with Hagopian et al. [13]. The trend towards a decrease between investigate and control groups (P=0.08) in our results could indicate a role for glycerol-3-phosphate dehydrogenase in the inter-conversion of glyceraldehyde to glycerol.

GAFD activity increase can be associated with an increase in the need of erythrocytes for ATP and an increase in the production of 2.3 diphosphoglycerates required for the entry of oxygen into tissues. A parallel decrease in G3D activity confirms the assumption that the cell needs more energy, which confirms the data obtained by Benner et al. [14]; the proper move in the oxy-hemoglobin dissociation bend incorporates increases in temperature, 2.3-bisphosphoglyceric acid, and certain hemoglobinopathies like sickle cell hemoglobin, sulfhemoglobin.

## CONCLUSIONS

Establishing phosphothrioses changes suggest excessive use of phosphothrioses for ATP, ADP, AMP production. It destroys the balance of the energetic and plastic potential in erythrocytes and lymphocytes among patients with inflammatory breast cancer, infiltrative stomach cancer, and infiltrative colorectal cancer. Moreover, glycerol-3-phosphate dehydrogenase and glyceraldehyde-3-phosphate dehydrogenase action indicators, as well as the content of adenyl phosphates in erythrocytes and lymphocytes, can be used as markers for the choice of treatment tactics, possible correction, and prognosis of the disease course.

Therefore, further research is needed to identify adequate methods in case of impaired energy exchange at the level of blood cellular elements in the types of cancer studied and other cancers.

## Personal thanks

We want to thank Professor Irina Yurievna Bagmut.
